# Bilateral Internal Carotid Artery Agenesis in an 11-Year-Old Middle Eastern Child: A Case Report and Literature Review

**DOI:** 10.7759/cureus.91813

**Published:** 2025-09-08

**Authors:** Yahya M Alassouli, Hadi A Helali, Ganga Rajapandian, Samar Almuntaser

**Affiliations:** 1 Pediatric Neurology, Al Jalila Children's Specialty Hospital, Dubai, ARE; 2 Diagnostic Radiology, Al Jalila Children's Specialty Hospital, Dubai, ARE

**Keywords:** headache, neuroradiology, pediatrics, vascular malformation, vascular neurology

## Abstract

Agenesis of the internal carotid artery (ICA) is an ultra-rare congenital anomaly. We report the case of an 11-year-old male patient who was referred to the pediatric neurology department for the assessment of left leg pain and intermittent, unspecified headaches for four years. On assessment, the left lower limb was wasted and shorter than the right one. Furthermore, he exhibited a complex collection of ocular diseases. Other examinations were unremarkable. Laboratory results were normal, and neuroimaging revealed a bilateral absence of the internal carotid artery. Nevertheless, the patient remained clinically stable, was not on any medications, and was regularly followed up for his comorbidities by a multidisciplinary team. This report highlights the various clinical manifestations of ICA agenesis and the need for a high clinical suspicion for early diagnosis and management. It also includes a review of the literature, highlighting the clinical aspects, embryological origin, and other reported cases.

## Introduction

Agenesis of the internal carotid artery (ICA) is a rare deformity, accounting for less than 0.01% of reported cases in the population [1]. It occurs when there is a congenital absence of the internal carotid artery, which can be unilateral or bilateral, with the latter being less frequent [2]. This defect arises due to abnormal regression of the first and third aortic arch, but the precise etiology remains unclear [2]. There is a variety of clinical manifestations of this condition, ranging from asymptomatic incidental findings to developmental delays, hypopituitarism, transient ischemic attack (TIA), and multi-organ malformations [3]. Complications can be serious and fatal. For instance, it has been shown that there is a significantly higher association of aneurysms seen in these patients compared to the general population, with increased risk of rupture, causing subarachnoid hemorrhage (SAH) [2]. This should prompt physicians to have high clinical suspicion and do early interventions to prevent critical sequelae. As there is no established standard of care for this condition, treatment is primarily focused on managing associated complications through regular follow-up, which enables early detection and intervention [3]. Moreover, diagnosis can be made through a brain magnetic resonance imaging (MRI), while a brain computed tomography (CT) also helps confirm the absence of the carotid canal [4]. Periodic follow-up of cerebral vasculature is recommended, preferably using magnetic resonance angiography (MRA) due to its non-invasive nature and lower risk of complications [5].

Herein, we report a case of a male child who presented with unspecified headaches and unilateral leg pain. He was later found to have bilateral ICA agenesis on brain magnetic resonance imaging (MRI). This is followed by a literature search to highlight the rare embryology, clinical manifestations, diagnostic modalities, and management approach of ICA agenesis.

## Case presentation

This is a case of an 11-year-old male patient referred to the pediatric neurology department for clinical assessment of left leg pain and headaches. The initial presentation was four years earlier, when the patient had the same complaints, but the parents did not seek medical attention at that time.

The boy was born at term through an elective cesarean section. He had a normal birth weight and an uneventful birth. He is the third among four children born to first-cousin consanguineous parents. On further questioning, the parents revealed that the child's younger sister has Peter's anomaly in the left eye. Simultaneously, one of his maternal aunts has been blind since birth, and another one died in her 20s due to systemic lupus erythematosus (SLE) complicated by renal failure. Additionally, two of the mothers' maternal cousins had intellectual disabilities. He achieved typical developmental milestones at appropriate ages, but he had specific learning difficulties resulting from his affected vision.

Neurological examination revealed that the child had dysmorphic features (a high-arched palate and a severely deviated nasal septum with hypertrophy). He also had two café-au-lait spots and pectus carinatum.

While walking, he displayed left-sided limping, especially after a long distance. On inspection, the left lower limb was wasted and shorter than the right lower limb. Moreover, on his motor assessment, there was spasticity and a brisk reflex in the left ankle. In addition, he also had left-sided spastic equinus and a positive Babinski reflex. That being said, his power was 5/5 in all limbs. Examinations of the rest of the extremities were normal.

Furthermore, the child was referred to the ophthalmology clinic for a detailed assessment of his vision and eyes. The ocular examination revealed iridocorneal endothelial syndrome, anterior segment dysgenesis, bilateral extraocular muscle weakness, congenital right eye cataracts, central corneal opacity of the right eye, ptosis of both eyelids, and nystagmus.

In addition, due to suspicion of an autoimmune or genetic cause for the child's underlying condition, a vasculitis workup was sent and returned negative. Simultaneously, genetic testing, including chromosomal microarray, followed by whole-exome sequencing, was sent and yielded irrelevant results to the clinical presentation.

**Figure 1:**
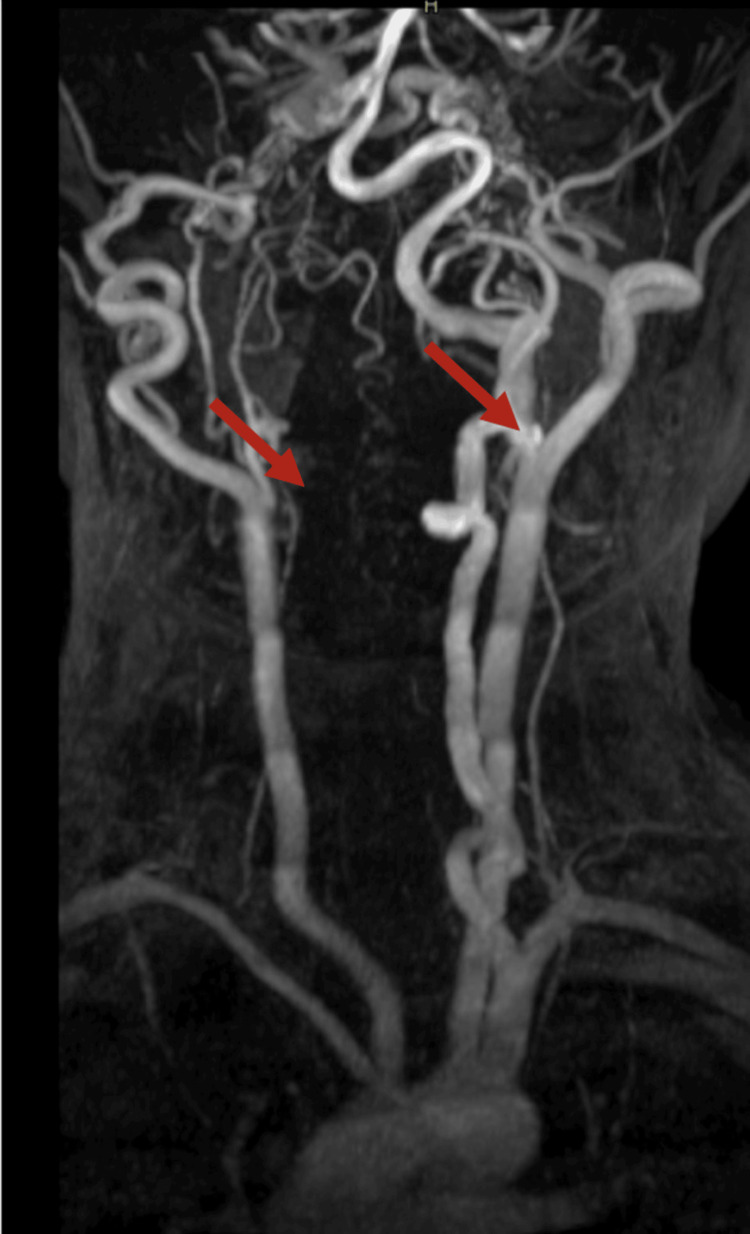
Coronal magnetic resonance angiogram of the neck showing bilateral absence of the ICA (red arrows indicate the usual place of the ICA) ICA: internal carotid artery

**Figure 2:**
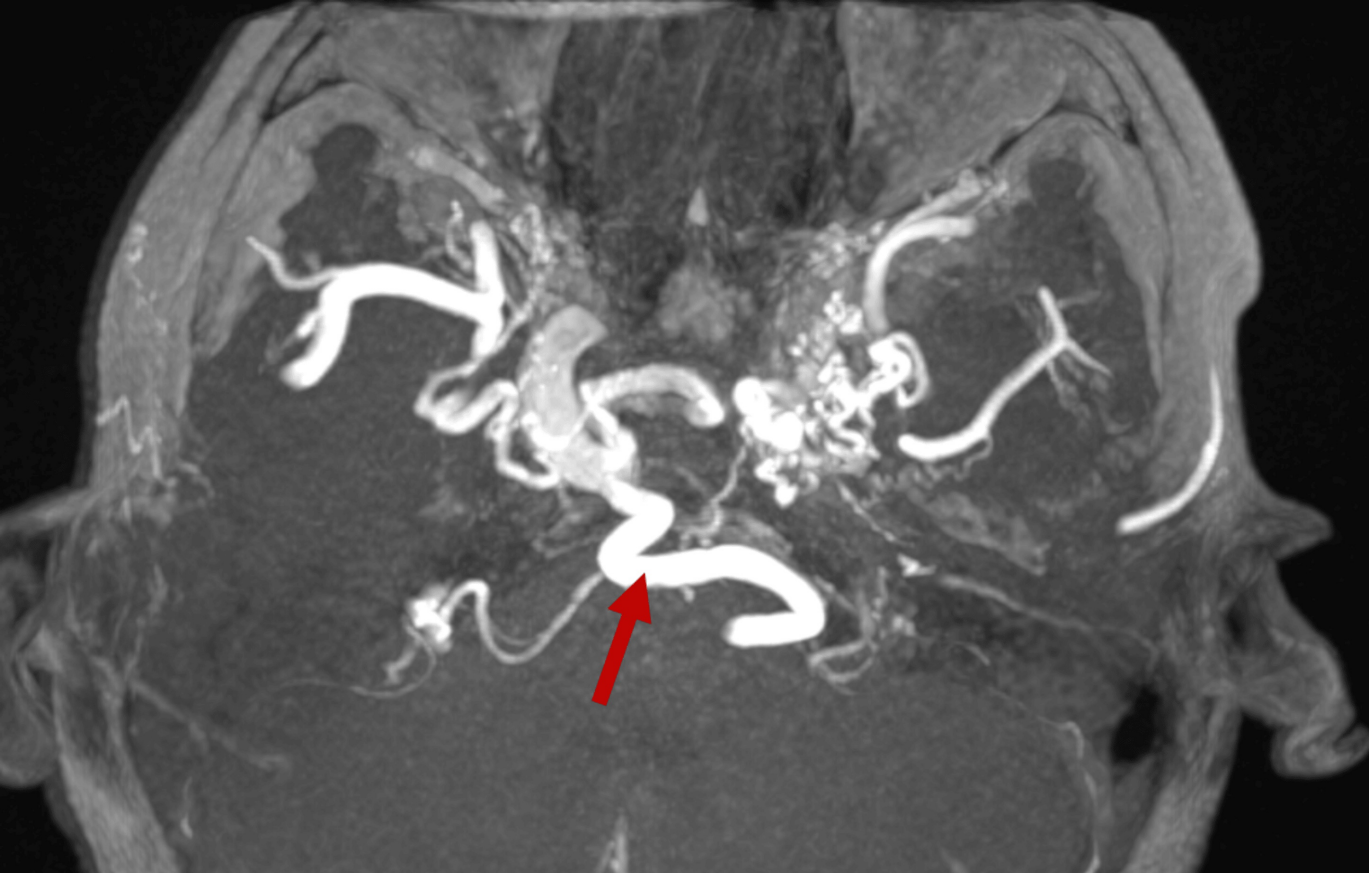
Axial magnetic resonance angiogram showing the intracavernous part of the right internal carotid artery reconstituted by a persistent trigeminal artery branching from the basilar artery (red arrow)

Concurrently, a brain and neck MRI with magnetic resonance angiography (MRA) and venography (MRV) was performed. The images revealed bilateral absence of the internal carotid artery. On the right, it is reformed by the persistent trigeminal artery from the basilar artery. Simultaneously, on the left, the intracranial circulation is maintained by the vertebrobasilar trunk through the persistent trigeminal artery, the collaterals from the circle of Willis, and transcranial collateral arteries from the branches of the external carotid artery (Figure 1 and Figure 2).

**Figure 3:**
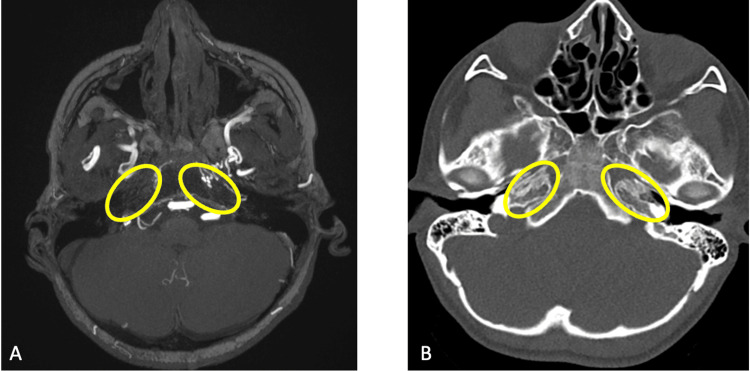
(A) Axial MRA showing absent petrous parts of both internal carotid arteries (yellow circles show ossified canals) and (B) axial CT showing bilateral absent (ossified) carotid canals (yellow circles) MRA: magnetic resonance angiography, CT: computed tomography

Retrospectively, a computed tomography (CT) scan of the head performed earlier for sinusitis assessment was reviewed and showed the absence of the internal carotid canals, confirming the MRI finding of bilateral agenesis of ICA and raising high suspicion for congenital etiology (Figure 3).

On regular follow-up for comorbidities with a multidisciplinary team of orthopedics, otolaryngology, ophthalmology, and physiotherapy, the patient remained clinically stable, with no worsening of his symptoms.

## Discussion

ICA agenesis is a rare congenital abnormality; according to the literature, fewer than 200 cases have been reported [2]. Several terms can be used interchangeably when describing an absent or an abnormally small ICA, such as agenesis, hypoplasia, and aplasia. However, the bony carotid canal is absent in agenesis, whereas it remains present but smaller in hypoplasia/aplasia [2]. An insult in early fetal life could lead to malformation of the ICA and thus ossification of the carotid canal [6]. Moreover, bilateral absence of the internal carotid artery (ICA) is much less common than unilateral absence, and its occurrence in pediatric patients is even rarer [7].

The ICA develops by week 5 of gestation, as a terminal branch from the common carotid artery (CCA) [6]. It is debatable whether it has the same embryological origin as the external carotid artery (ECA) and/or the CCA [6]. The current case report presents findings that support the theory that they have different embryologies, as evidenced by the absence of the ICA in this patient, whereas the ECA and CCA remained patent.

Several reports have shown that most cases remain asymptomatic because of the formation of an effective collateral system that supplies cerebral circulation [2,6,7]. A study by Zhang et al. (2018) revealed a correlation between the clinical manifestations of ICA agenesis and age [3]. In that study, young patients presented with developmental delay, subarachnoid hemorrhage, or other developmental abnormalities in multiple organs, whereas older patients mainly presented with transient neurological attacks [3]. This may be explained by the fact that, in elderly individuals, intracranial collateral circulation becomes decompensated due to atherosclerosis, leading to transient ischemic attacks (TIA). In contrast, younger individuals often develop effective compensatory intracranial hemodynamic changes through collateral pathways, which can increase the risk of aneurysm formation and subsequent rupture, leading to subarachnoid hemorrhage (SAH) [3]. Nevertheless, a paper by Alhaizaey et al. (2020) has revealed that ICA agenesis could also occur in the younger generations as a TIA [6]. Still, less common presentations such as headaches and Horner's syndrome exist [1,7].

Most absent ICA cases were diagnosed incidentally after a brain MRI was performed for an unrelated complaint [4]. However, after the initial diagnosis by MRI, a head CT scan should be performed to confirm the absence or hypoplasia of the carotid canal [4]. That being said, bilateral ICA agenesis can be associated with other conditions such as cerebral autosomal dominant arteriopathy with subcortical infarcts and leukoencephalopathy (CADASIL) usually discovered in adults, as reported by MacDonald and Alvaro (2020) [8], or sickle cell disease (SCD), as documented by Markovic et al. (2022) [9] in a five-year-old child with stroke.

Given the congenital and asymptomatic nature of carotid agenesis, no definitive treatment is available, nor required [3]. Despite that, a thorough evaluation for associated intracranial and extracranial aneurysms is essential, given their increased vascular risk [5]. Cardiovascular risk factors must be rigorously managed through blood pressure control, lipid regulation, and lifestyle counseling. In addition to that, a prompt evaluation of any neurological symptoms is critical, and head and neck surgeries carry significant risk due to altered cerebral circulation [5]. In the current case, the patient received supportive management through a multidisciplinary approach.

In a study by Xu et al. (2022), five patients with bilateral internal carotid artery agenesis were evaluated to investigate the cerebrovascular complications associated with ICA agenesis [10]. Digital subtraction angiography (DSA) revealed intracranial aneurysms in three patients, a dural arteriovenous fistula in one, and a rete aneurysm in another. Three patients underwent endovascular intervention, while one was treated with bypass surgery. Notably, no cerebrovascular accidents or mortality were reported during short-term follow-up [10]. Another research by Vasović et al. (2017) has found that the percentage of aneurysms reached 11.6% of reported cases [11].

It is recommended that patients undergo periodic cerebral vessel assessments, with magnetic resonance angiography (MRA) preferred over other modalities due to its non-invasive nature and lower risk of complications [5].

Table 1 summarizes the clinical findings and outcomes in patients with ICA agenesis reviewed in the literature [1-11].

**Table 1:** Comparative summary of reported cases and reviews on congenital ICA agenesis/hypoplasia ICA: internal carotid artery, ACom: anterior communicating artery, PCom: posterior communicating artery, ECA: external carotid artery, MCA: middle cerebral artery, ACA: anterior cerebral artery, US: ultrasound, CTA: CT angiography, MRA: MR angiography, DSA: digital subtraction angiography, SAH: subarachnoid hemorrhage, TIA: transient ischemic attack, DAVF: dural arteriovenous fistula, EC-IC: extracranial-intracranial bypass, BP: blood pressure, CADASIL: cerebral autosomal dominant arteriopathy with subcortical infarcts and leukoencephalopathy

Author (year)	Demographics and associated conditions	Laterality	Presentation	Diagnostics	Collateral pathways	Management	Outcomes
Whitley et al. (2022) [4]	Systematic review (41 papers), 50 patients	Mostly unilateral	Asymptomatic, headache, vertigo, TIAs	CTA/MRA, Doppler, DSA	ACom/PCom, rare ECA-ophthalmic	Conservative, BP control, aneurysm surveillance	Generally favorable, high stroke/aneurysm risk
Markovic et al. (2022) [9]	5 years, male, sickle cell disease	Bilateral	Ischemic stroke, seizures	MRI, MRA, CTA	Basilar + PComs	Exchange transfusion, anticonvulsant, transfusions	Mild hemiparesis, no recurrence
Xu et al. (2022) [10]	5 adults (30-65 years)	3 bilateral, 2 unilateral	Headache, SAH, DAVF, dizziness	DSA, CTA/MRA, skull base CT	Basilar + PComs, carotid rete, ECA collaterals	Bypass + coil, embolization, conservative	Good short-term, stable follow-up
MacDonald and Alvaro (2021) [8]	41 years, male, CADASIL	Bilateral	Orthostatic dizziness, progressive decline	CTA, MRI, skull base CT, genetics	Basilar + PComs	Conservative, CADASIL management	Lost to follow-up, no ICA stroke on discharge
Alhaizaey et al. (2020) [6]	6 years, female, healthy	Bilateral	Ischemic stroke, seizures	MRI/CTA, MRA	Basilar + PComs, carotid rete mirabile	Acute stroke management, aspirin	Full recovery, stroke-free for 6 months during follow-up
Zhang et al. (2018) [3]	64 patients (5 months-75 years)	Mostly unilateral (13 bilateral)	TIAs, SAH, seizures, headaches, incidental	CTA/MRA, DSA, CT	Circle of Willis (ACom, PCom), ECA-ophthalmic	Conservative, aneurysm clipping/coiling in some	Good with collaterals, aneurysms in 25%
Vasović et al. (2018) [11]	Literature review (60 cases)	Bilateral	Variable: asymptomatic, stroke, SAH, delay	CTA/MRA, skull base CT, DSA	Circle of Willis, vertebrobasilar, ECA collaterals	Conservative, aneurysm repair, EC-IC bypass	25% asymptomatic, aneurysm ~12%, variable
Perla et al. (2017) [1]	12 years, male, healthy	Unilateral (left)	Headache, transient amaurosis fugax	Doppler US, CTA, MRA	ACom cross-flow, intercavernous anastomosis	Conservative, annual MRA	Stable for 7 years, no aneurysm
Li et al. (2017) [2]	15 years, female, healthy	Unilateral (right)	Asymptomatic, seizures unrelated	CTA/MRA, DSA, skull base CT	ACom (ACA), PCom (MCA)	Conservative aneurysm surveillance	Neurologically intact
Khan et al. (2012) [7]	16 years, male, healthy	Unilateral (left)	Horner's syndrome, headaches	MRI/MRA, DSA, skull base CT	ACom (ACA), PCom (MCA), ECA-ophthalmic	Conservative	Stable, persistent Horner's
Steer and Rowe (2008) [5]	13 years, male, healthy	Bilateral	Thunderclap headaches	MRI/MRA, skull base CT	Vertebrobasilar + PComs, ECA-ophthalmic	Conservative, stroke prevention	No stroke, stable follow-up

## Conclusions

Internal carotid artery (ICA) agenesis is an exceptionally rare congenital anomaly, often discovered incidentally during imaging for unrelated complaints. This condition is usually asymptomatic due to robust collateral circulation; however, clinical manifestations can vary with age and may include transient ischemic attacks, developmental delays, or subarachnoid hemorrhage. Accurate diagnosis relies on both MRI and confirmation with CT to assess the presence or absence of the bony carotid canal, distinguishing agenesis from hypoplasia. While no standardized treatment exists, management is typically supportive and tailored to the individual's clinical presentation, with surgical intervention considered for complications such as aneurysms. This case emphasizes the importance of recognizing this anomaly early and highlights the need for a multidisciplinary approach to ensure appropriate follow-up and care. Finally, the embryological origin of the ICA is postulated to differ from those of the CCA and ECA, as evident in this patient and other cases reviewed in the literature.
